# The efficacy of minocycline hydrochloride ointment versus iodoform gauze for alveolar osteitis: A prospective cohort study

**DOI:** 10.1186/s12903-022-02468-9

**Published:** 2022-10-18

**Authors:** Yu-qi Sun, Rui Sun, Ji-hong Zhao

**Affiliations:** 1 The State Key Laboratory Breeding Base of Basic Science of Stomatology (Hubei-MOST) & Key Laboratory of Oral Biomedicine Ministry of Education, School & Hospital of Stomatology, Wuhan University, No. 237 Luoyu Road, 430079 Wuhan, China; 2 Department of Oral and Maxillofacial Surgery, School & Hospital of Stomatology, Wuhan University, No. 237 Luoyu Road, 430079 Wuhan, China

**Keywords:** Alveolar osteitis, Therapeutics, Minocycline, Iodoform gauze

## Abstract

**Background:**

Alveolar osteitis (AO) is one of the most commonly encountered complication following tooth extraction, however, to date there is no standard methods of prevention and treatment. The study aims to investigate the efficiency of minocycline hydrochloride ointment (MHO) for the treatment of alveolar osteitis compared with traditional treatment with iodoform gauze (IG).

**Methods/design:**

STROBE checklist was followed to report this study. All patients underwent tooth extraction either in our department or other hospitals, whom presented with postoperative pain, were screened out to meet the inclusion and exclusion criteria of this study about AO. Patients who fulfilled the inclusion criteria were enrolled in our prospective cohort study, and MHO or IG was administered. The Visual analog scale scores were used to assess the pain score of patients. The healing status of the extraction sockets was followed up. Differences in responses between groups were analyzed using Mann-Whitney U tests. Chi-square test was performed to explore the differences in the teeth position of AO.

**Results:**

Of 41,371 patients underwent tooth extraction with post-operative follow-up in our departments, only 20 patients (0.05%) suffered from AO. 31 patients with AO, whose teeth were extracted in other places, were also enrolled. The incidence of AO was significantly higher in third molars than other teeth (*P* < 0.01). In 28 patients that were treated with MHO, the pain was relieved substantially on day 3 and almost painless on day 7. And only 25% of cases required dressing change more than once. Whilst 23 patients treated with IG, the pain was relieved on day 5, and 56.5% of cases required multiple dressing change. The difference between the two groups of VAS scores had statistical significance during treatment at 8 h, 24 h, 3d, 5d, and 7d. No allergic reaction or further infection occurred.

**Conclusion:**

MHO has a safer and higher therapeutic effect in the treatment of AO compared with traditional treatment with IG. MHO may become a preferred treatment modality for AO.

**Supplementary information:**

The online version contains supplementary material available at 10.1186/s12903-022-02468-9.

## Introduction

Alveolar Osteitis (AO) is a painful complication after tooth extraction, also known as dry socket, localized osteitis, and fibrinolytic alveolitis [1]. Blum described AO as the manifestation of “postoperative pain in and around the extraction site, which increases in severity at any time between 1 and 3 days after the extraction accompanied by a partially or totally disintegrated blood clot within the alveolar socket with or without halitosis” [2]. The incidence of AO after all extractions is about 1–5% worldwide, mostly in posterior mandibular teeth, and up to 38% of lower third molar extractions [3].

Some prophylactic methods, such as chlorhexidine mouthwash, antibiotics, and surgical techniques showed reduced incidences of AO [1]. Although a wide array of reported data covered AO, there is no standard prevention and treatment to date [1]. The treatment principle of AO is to thoroughly debride and isolate the external stimulation to the alveolar bone, to achieve rapid hemostasis and promote healing. The conventional treatment for AO involves sterile saline irrigation of the socket until all visible debris has been eliminated, followed by the placement of an obtundent dressing into the socket [1]. Some studies reported the use of different irrigants to rinse the alveolar socket such as clindamycin, rifampicin [4], or fill it with medicaments that include alvogyl, zinc-oxide eugenol (ZOE) [5, 6], honey [7], turmeric [8], and Holisal gel [9]. Nevertheless, these methods can only alleviate the symptoms but do not enhance the healing of the socket [10]. Some recent regenerative wound-healing technologies in AO such as platelet-rich fibrin (PRF) [11], concentrated growth factor (CGF) [12, 13], and low-level laser therapy (LLLT) [10] have shown effectiveness. However, LLLT incurs a higher cost and it is technically challenging to find the optimal dosage for a particular therapy [10].

Minocycline hydrochloride ointment (MHO) is a semi-synthetic and high-efficiency tetracycline. Minocycline is considered a broad-spectrum antibiotic because it is active against a wide range of aerobic and anaerobic gram-positive and gram-negative bacteria, and other microorganisms [14]. MHO is applied locally into pockets utilizing a syringe applicator in a biodegradable controlled-release system [15]. MHO has been widely used in management of periodontitis [16] and peri-implantitis [17], however, the application in AO is yet to be reported. In this study, we aim to investigate the efficacy of MHO in the treatment of AO compared with traditional treatment with IG.

## Materials and methods

**Fig. 1:**
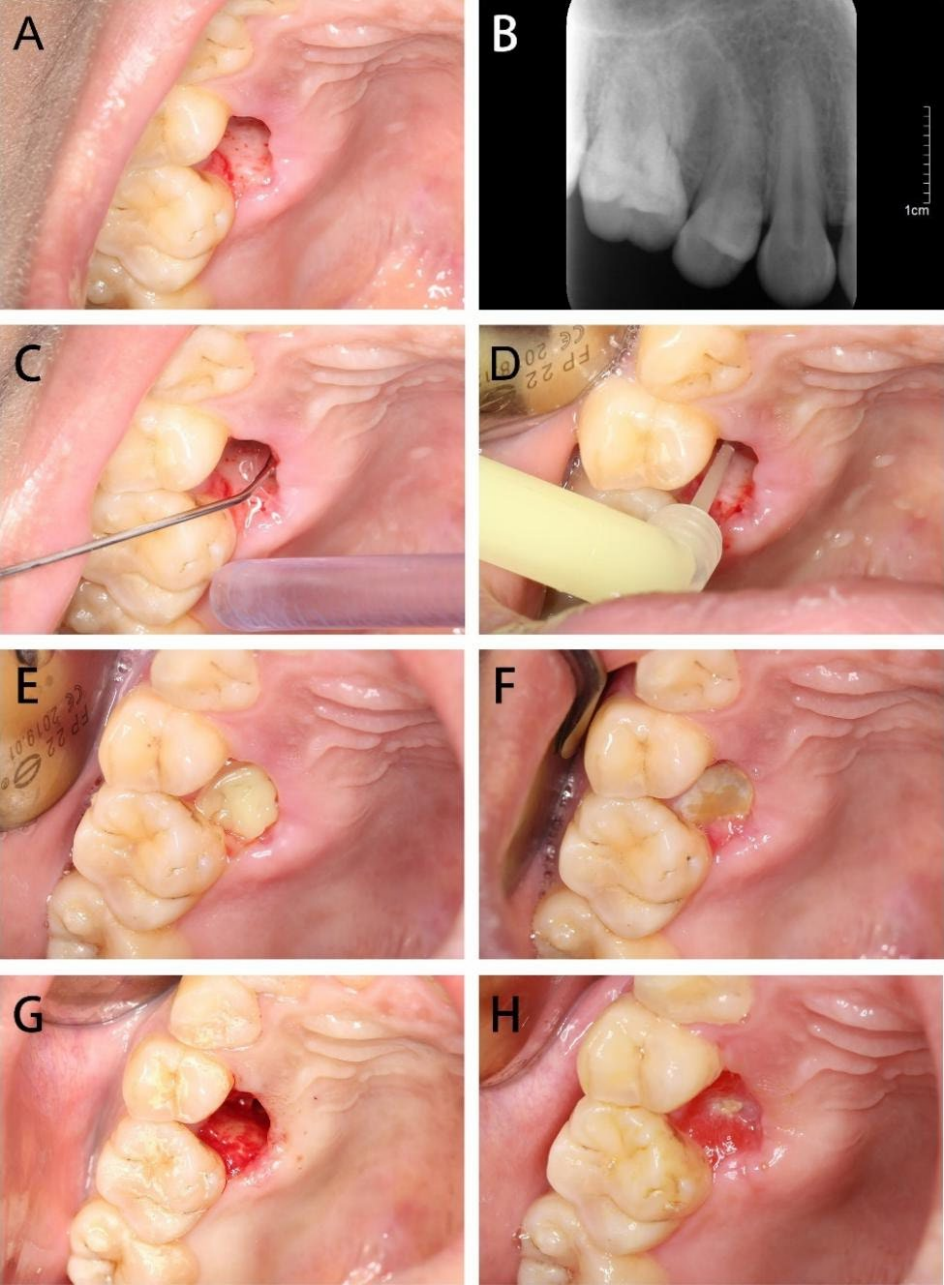
The process of treatment with MHO for AO: A, Preoperative clinical view of AO in right maxillary second premolar region; B, x-ray image; C, After alveolar irrigation of socket with saline; D-E, Placement of MHO; F-G, Postoperative 5th day, MHO was still attached to the alveolar bone (F), and granulation tissue formation was observed in the socket after irrigation (G); H, Postoperative 20th day, the alveolar socket was healed, and a small amount of MHO remained in it

STROBE checklist was followed to report this study. This study followed the Declaration of Helsinki on medical protocol and ethics and obtained the approval by the regional Ethical Review Board of the Ethics Committee of the Hospital of Stomatology, Wuhan University (No.2021-B34). Patients who underwent dental extractions either within the department or elsewhere, were screened for the study. During this period, all patients whose teeth were extracted in our department were recorded, and follow-up visits by telephone and short messages were carried out to advise for review if presented with discomfort. During the review, patients were enrolled in the prescreen procedure. Patients, whose teeth were extracted in other hospitals, were also enrolled in the prescreen procedure if they were referred to our department for postoperative pain. In the prescreen procedure, the signs and symptoms were assessed simultaneously by 3 senior physicians for diagnosis of AO. Patients aged between 18 and 70, who were diagnosed with AO were included in the study. AO was diagnosed according to a standard criterion, in which the postoperative pain was present in and around the extraction site, that increased in severity at any time between 1 and 3 days after the extraction accompanied by a partially or totally disintegrated blood clot within the alveolar socket with or without halitosis [2]. The exposure of alveolar bone happened when the blood clot dissolved [18](Fig. 1 A). Radiologic examination showed no residual root in the alveolar socket (Fig. 1B). Other possibilities that may cause post-operative pain, such as residual root, maxillofacial space infection and systemic infection after tooth extraction and other painful symptoms were ruled out. Patients who refused our MHO treatment, who smoked, with systematic diseases or mental disorders, or pregnant and breastfeeding women were also excluded.

Based on the inclusion and exclusion criteria described above, 51 patients diagnosed with AO were enrolled. According to different treatment methods, patients were divided into MHO Group and IG Group. The population of MHO Group was 28, and the population of IG Group was 23. The treatment procedures in both groups were standardized and performed by the same team of doctors.

MHO Group: Once the patients were recruited and signed the treatment consent, copious irrigation of the extraction socket with saline was performed under local anesthesia (Fig. 1 C). Debris was removed from the alveolar socket gently if needed. The procedure, repeated curettage of the socket to create fresh clot is avoided. After debridement, MHO was applied to the alveolar socket using the syringe. The nozzle was placed at the base of the alveolar socket to inject the MHO into the socket gently (Fig. 1D and E).

IG Group: Similarly, patients were recruited and signed the treatment consent. Copious irrigation of the alveolar socket was done with saline under local anesthesia. Debris was removed from the alveolar socket gently if needed. Then IG was applied locally in the alveolar socket.

The medical records and imaging data of all patients with AO were collected. The treatment process of AO was recorded, including materials, drugs, and methods. Patient’s pain scores were recorded using a visual analog pain scale (VAS) from 0 to 10 before and after treatment for AO. The duration of pain relief and the number of dressing changes at follow-up visits were also recorded. The clinical outcome and endpoint of the study was pain relief and mucosal healing.

The statistical analysis was conducted by SPSS 26.0, and the statistical chart was conducted by GraphPad Prism 8, and a *P*-value of < 0.05 was considered statistically significant. Chi-square test served to explore the differences in the teeth position of AO, doctors, and time of dressing changes. Mann-Whitney U test was used for comparison of VAS scores between the two groups.

## Results

**Fig. 2:**
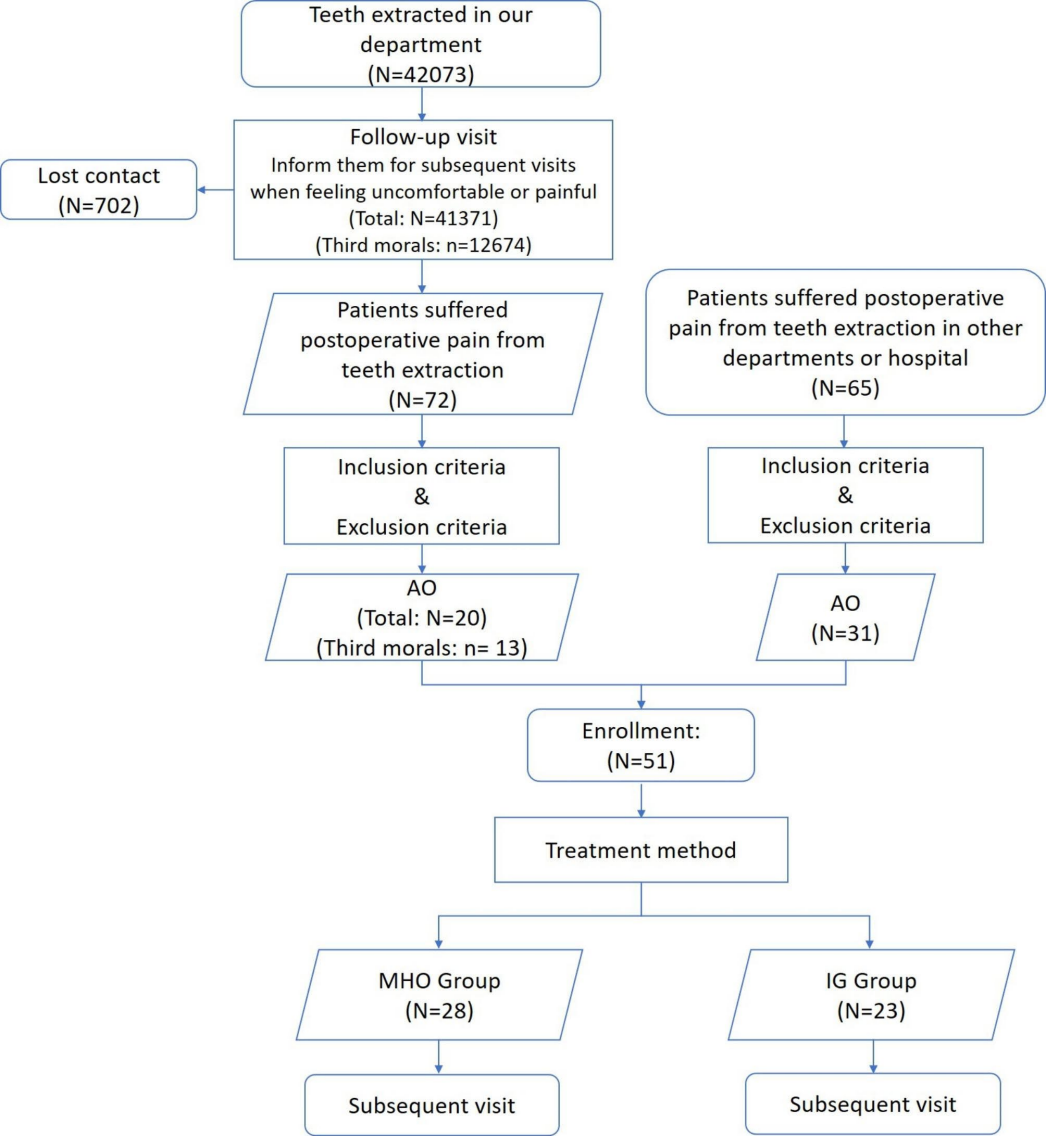
The flow diagram for the included studies

The study was conducted from August 2021 to January 2022 in the outpatient clinic of Department of Oral and Maxillofacial Surgery, Hospital of Stomatology, Wuhan University. A total of 42,073 teeth were extracted in our department. 702 patients lost to follow-up. Of the 41,371 patients’ follow-ups, 12,674 were third molars. 72 patients were recalled to our department due to severe post-extraction pain, and 20 of them were diagnosed as AO. Among them, 13 were third molars. 31 patients presented with post-extraction pain, whose teeth were extracted in other places, were also diagnosed as AO (Fig. 2).

To sum up, 51 patients with AO were enrolled in this study. There were 21 males and 30 females (41.2% and 58.8%, respectively) with a mean age of 36.31 ± 14.15. Most of the teeth were third molars (37, 72.5%), followed by mandibular first molars and mandibular second molars (Table 1). In our department, the incidence of AO was as low as 0.05% (20/41,371), and the incidence of AO in third molars was 0.10% (13/12,674) (Fig. 2). The incidence of AO was significantly higher in third molars than other teeth (*P* < 0.01).

**Table 1:** Clinical characteristics of 51 AO patients

		Total			MHO Group			IG Group	
		N = 51			N = 28			N = 23	
		Case number	%		Case number	%		Case number	%
**Gender**									
	Male	21	41.2%		11	39%		10	43.5%
	Female	30	58.8%		17	61%		13	56.5%
**Age**									
	<35	31	60.8%		16	57%		15	65.2%
	≥ 35	20	39.2%		12	43%		8	34.8%
**Tooth position**									
	Third molars	37	72.5%		18	64%		19	82.6%
	Other teeth	14	27.5%		10	36%		4	17.4%
**Dressing times**									
	= 1	34	66.7%		21	75%		13	56.5%
	> 1	21	41.2%		7	25%		10	43.5%

**Fig. 3:**
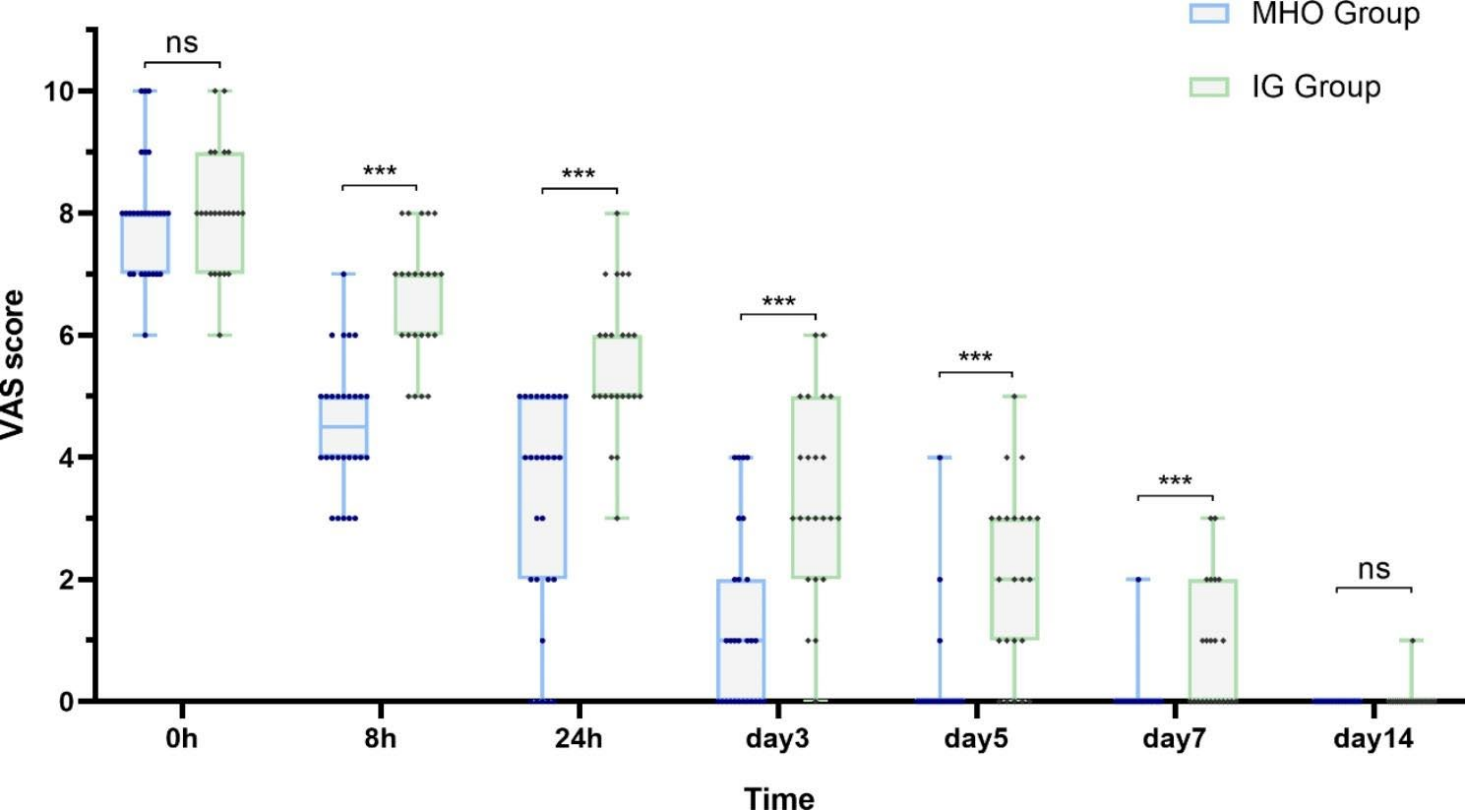
VAS scores of patients in both groups after treatment

Visual analog scale (VAS) scores were used to assess the pain score of patients. The patients in the MHO Group felt severe pain (pain scores ranged from 6 to 10) on their first visit for AO treatment, and the pain was greatly relieved (pain scores less than 3) on day 3 and closed to entirely painless on day 7. Only 25% of patients had dressing changes more than once. In contrast, most patients in IG Group experienced considerable pain relief mainly on day 5, and 43.5% of patients had dressing changes more than once (Supplementary Tables 1, 2). The difference between two groups of VAS scores had statistical significance during treatment at 8 h, 24 h, 3d, 5d, and 7d. The efficacy of the MHO Group is noticeably better than the IG Group (Fig. 3). The most common adverse reaction was encountered at the first time of MHO administration, where 4 patients experienced severe pain for several hours after the local anesthesia wears off, however the pain was relieved subsequently. No allergic reaction or further infection occurred (Supplementary Table 3). The patients were asked to return to the clinic 3–5 days later, and in MHO Group, MHO was still present in the alveolar socket, attached to the alveolar bone (Fig. 1 F). Granulation tissue formation was observed in the socket after irrigation (Fig. 1G). All of the alveolar sockets were healing, and the dressings were changed not more than 3 times (Fig. 1 H).

## Discussion

The main symptom of AO is pain, therefore the treatment should aim to resolve the pain as soon as possible. Our study found that, compared with conventional treatment with IG, MHO can relieve the pain rapidly without frequent follow-up visits. In the sockets treated with MHO, a low VAS score of 2–4 was obtained as early as day 2, and most patients achieved significant pain relief within 8 h after treatment. We also found that the patient’s alveolar socket was healing in the review visit. As the bone surface was still covered with MHO, the dressing change did not exceed more than 3 times. This observation demonstrated that the use of MHO in the treatment of AO can achieve good results. To our knowledge, this is the first article describing the usage of MHO in AO.

At present, the etiology of AO is considered to be comprehensive and multifactorial. Risk factors include fibrinolysis (destruction of the clot), bacteria, trauma, smoking, medications, and anatomic considerations [1]. In recent years, due to the application of minimally invasive technology, the use of high-speed air turbines, piezosurgery, and other tools can shorten the time of tooth extraction by removing bone, and sectioning crown in higher efficiency. More importantly, it does not crush the bone tissue and does not damage the blood supply of alveolar bone. The popularization of minimally invasive technology is gradually replacing the traditional chisel method, and the incidence of AO has been significantly reduced in China. According to the clinical observation in our hospital, the incidence of AO in third molars is about 0.10%. Due to the expertise of our hospital in the region, some patients who experienced postoperative pain following tooth extraction would opt to come to our hospital for subsequent treatment even though the extraction was previously done at other hospitals.

MHO is a well-known sustained release local drug delivery system in management of periodontitis, which forms a hard layer of the membrane when in contact with water, allowing slow release of the drug [19]. At the follow-up visit, we found that MHO was firmly adhered to the alveolar fossa, which protected the alveolar bone from external stimulus and facilitated the formation of granulation tissue in the alveolar bone.

Shen et al. [20] used high-throughput sequencing analysis of microbial profiles (16 S rRNA gene), and found that *Parvimonas*, *Peptostreptococcus*, *Fusobacterium*, and *Prevotella* species are considered the key population that could have crucial roles in the pathogenesis or maintenance of AO. Based on the result, we hypothesized that MHO, as a broad-spectrum antibiotic, could be used effectively in the treatment of AO.

Dissolution of the blood clot is one of the most characteristic features in AO, which seems to be explained by the release of tissue activators in the alveolar bone and subsequent dissolution of the blood clot by the action of plasmin, which is formed by the activation of plasminogen in the clot [21]. Minocycline is a neuroprotective agent that inhibits proteolytic enzymes and therefore could potentially both inactivate the clot lysis effect and decrease the damaging effects of tissue plasminogen activator [22]. Minocycline also inhibited the expression of cell surface markers of M1-polarized microglia (CD86 and CD68) as well as the production of inflammatory cytokines (IL-1b, TNF-a, and IFN-g) in vivo and in vitro [23]. Microglia and astrocytes act as possible modulators of neuropathic pain by releasing some cytokines and chemokines [24]. Preemptive and repeated systemic administration of minocycline attenuates the development of neuropathic pain symptoms [25]. This may partly explain the use of MHO in relieving pain in patients with AO.

Several clinical studies have been conducted to improve the efficacy of AO, including drug irrigation, material packing, and photobiomodulation therapy. Çebi [4] found that intra-alveolar irrigation with antibiotics was effective in pain and alveolar mucosa healing in the treatment of alveolar osteitis. In that study, intra-alveolar irrigation was applied with sterile saline, clindamycin, and rifampicin-containing antibiotics every 2 days for 10 days. This treatment required repeated visits, and the pain score dropped to 4 and below till day 5. Supe et al. [5] concluded that alvogyl (Combination of Iodoform + Butylparaminobenzoate) was the most successful combination for the management of AO, and ZOE was a cost-effective and easily available medicament for dressing. The study showed the mean time required for complete pain relief in the alvogyl group was 6.52 ± 1.88 days and in the ZOE group, it was 9.06 ± 2.14 days. The treatment required more than 4 dressing changes. Kamal et al. [13] found that CGF and LLLT had superiority in enhancing alveolar socket wound healing compared with the conventional technique concerning reducing inflammation, producing granulation tissue (GT), and relieving pain. However, laser technology is costly and the need to adhere to Laser Protection Protocol may be an obstacle to providing this armamentarium in all surgical practice [13]. Moreover, it’s difficult to find the optimal therapeutic dosage [10]. While in autologous plasma, it has been shown that PDGF, TGFβ1, VEGF and EGF cytokines were all significantly greater in platelet-rich plasma samples than in the whole blood baseline samples [26]. CGF, as the third generation of autologous plasma, was shown to promote cell proliferation, migration, and angiogenesis process [27]. Autologous CGF is safe to use without immunological rejection. Its preparation technique is relatively simple and the processing work for CGF production can be conducted at the chairside in the dental office or oral surgery suite at an affordable cost [13]. However, CGF requires at least 9 mL of blood, and the extra CGF processing time needed may impose time constraints in a busy practice [13]. And CGF requires specific equipment, which is not widely available in China. MHO is a straightforward, economical and practical dressing in management of AO.

In this study, following the copious irrigation with saline and removing debris and necrotic material, MHO was placed in the socket. This treatment method is simple and easy to operate without having to curette of the alveolar socket repeatedly. For some patients with impaired healing of the alveolar socket, CGF can be subsequently introduced to promote wound healing.

## Conclusion

MHO has a safer and higher therapeutic effect in the treatment of AO compared with traditional treatment with IG. MHO may become a preferred treatment modality for AO.

## Electronic supplementary material

Below is the link to the electronic supplementary material.

Supplementary Tables

## Data Availability

All data generated or analyzed during this study are included in this published article [and its supplementary information files].

## Abbreviations

AO: alveolar osteitis.
MHO: minocycline hydrochloride ointment.
IG: iodoform gauze.
ZOE: zinc-oxide eugenol.
CGF: concentrated growth factor.
LLLT: low-level laser therapy.
PRF: platelet-rich fibrin.
VAS: Visual analog scale.
GT: granulation tissue.

## References

[1] Chow O, Wang R, Ku D, Huang W (2020). Alveolar Osteitis: A Review of Current Concepts. J oral maxillofacial surgery: official J Am Association Oral Maxillofacial Surg.

[2] Blum IR (2002). Contemporary views on dry socket (alveolar osteitis): a clinical appraisal of standardization, aetiopathogenesis and management: a critical review. Int J Oral Maxillofac Surg.

[3] Mamoun J (2018). Dry Socket Etiology, Diagnosis, and Clinical Treatment Techniques. J Korean Association Oral Maxillofacial Surg.

[4] Çebi AT (2020). Evaluation of the effects of intra-alveolar irrigation with clindamycin, rifampicin and sterile saline in alveolar osteitis treatment. J stomatology oral maxillofacial Surg.

[5] Supe NB, Choudhary SH, Yamyar SM, Patil KS, Choudhary AK, Kadam VD (2018). Efficacy of Alvogyl (Combination of Iodoform + Butylparaminobenzoate) and Zinc Oxide Eugenol for Dry Socket. Annals of maxillofacial surgery.

[6] Shafaee H, Bardideh E, Nazari MS, Asadi R, Shahidi B, Rangrazi A (2020). The effects of photobiomodulation therapy for treatment of alveolar osteitis (Dry Socket): Systematic review and meta-analysis. Photodiagn Photodyn Ther.

[7] Ansari A, Joshi S, Garad A, Mhatre B, Bagade S, Jain R (2019). A Study to Evaluate the Efficacy of Honey in the Management of Dry Socket. Contemp Clin Dent.

[8] Lone PA, Ahmed SW, Prasad V, Ahmed B (2018). Role of turmeric in management of alveolar osteitis (dry socket): A randomised clinical study. J oral biology Craniofac Res.

[9] Zorina OA, Petrukhina NB, Boriskina OA (2019). [Alveolar osteitis treatment using Holisal gel]. Stomatologiia.

[10] Kamal A, Salman B, Ar NH, Samsudin AR (2021). Management of dry socket with low-level laser therapy. Clin Oral Invest.

[11] Yüce E, Kömerik N (2019). Potential effects of advanced platelet rich fibrin as a wound-healing accelerator in the management of alveolar osteitis: A randomized clinical trial. Niger J Clin Pract.

[12] Kamal A, Salman B, Abdul Razak NH, Qabbani AA, Samsudin AR: The Efficacy of Concentrated Growth Factor in the Healing of Alveolar Osteitis: A Clinical Study. *International journal of dentistry* 2020, 2020:9038629. 10.1155/2020/9038629 PMC7240629 32454827

[13] Kamal A, Salman B, Razak NHA, Samsudin ABR (2020). A Comparative Clinical Study between Concentrated Growth Factor and Low-Level Laser Therapy in the Management of Dry Socket. Eur J Dent.

[14] Garrido-Mesa N, Zarzuelo A, Gálvez J (2013). Minocycline: far beyond an antibiotic. Br J Pharmacol.

[15] Jones AA, Kornman KS, Newbold DA, Manwell MA (1994). Clinical and microbiological effects of controlled-release locally delivered minocycline in periodontitis. J Periodontol.

[16] Javed S, Kohli K (2010). Local delivery of minocycline hydrochloride: a therapeutic paradigm in periodontal diseases. Curr Drug Deliv.

[17] Park S, Song Y, Cha J, Lee J, Kim Y, Shin H, Lee D, Lee J, Kim C. Adjunctive use of metronidazole-minocycline ointment in the nonsurgical treatment of peri-implantitis: A multicenter randomized controlled trial. Clinical implant dentistry and related research 2021. 10.1111/cid.13006 34139047

[18] Hamad SA, Naif JS, Abdullah MA (2016). Effect of Diode Laser on Healing of Tooth Extraction Socket: An Experimental Study in Rabbits. J Oral Maxillofac Surg.

[19] Shao W, Xiao F, Xu ZX, Ren RH, Wang Y, Wu YQ (2018). Treatment of severe periodontic-endodontic combined lesions with minocycline hydrochloride ointment combined with mineral trioxide aggregate. Experimental and therapeutic medicine.

[20] Shen LH, Xiao E, Wang EB, Zheng H, Zhang Y (2019). High-Throughput Sequencing Analysis of Microbial Profiles in the Dry Socket. J oral maxillofacial surgery: official J Am Association Oral Maxillofacial Surg.

[21] Cardoso CL, Rodrigues MT, Ferreira Júnior O, Garlet GP, de Carvalho PS (2010). Clinical concepts of dry socket. J oral maxillofacial surgery: official J Am Association Oral Maxillofacial Surg.

[22] Machado LS, Sazonova IY, Kozak A, Wiley DC, El-Remessy AB, Ergul A, Hess DC, Waller JL, Fagan SC (2009). Minocycline and tissue-type plasminogen activator for stroke: assessment of interaction potential. Stroke.

[23] Kobayashi K, Imagama S, Ohgomori T, Hirano K, Uchimura K, Sakamoto K, Hirakawa A, Takeuchi H, Suzumura A, Ishiguro N (2013). Minocycline selectively inhibits M1 polarization of microglia. Cell Death Dis.

[24] Zhuo M, Wu G, Wu LJ (2011). Neuronal and microglial mechanisms of neuropathic pain. Mol Brain.

[25] Mika J, Osikowicz M, Makuch W, Przewlocka B (2007). Minocycline and pentoxifylline attenuate allodynia and hyperalgesia and potentiate the effects of morphine in rat and mouse models of neuropathic pain. Eur J Pharmacol.

[26] Lubkowska A, Dolegowska B, Banfi G (2012). Growth factor content in PRP and their applicability in medicine. J Biol Regul Homeost Agents.

[27] Jin R, Song G, Chai J, Gou X, Yuan G, Chen Z (2018). Effects of concentrated growth factor on proliferation, migration, and differentiation of human dental pulp stem cells in vitro. J tissue Eng.

