# Supplementary material for: The efficacy of minocycline hydrochloride ointment versus iodoform gauze for alveolar osteitis: A prospective cohort study
Source: BMC Oral Health. 2022 Oct 18;22:448. doi: 10.1186/s12903-022-02468-9 (PMC9580180; doi:10.1186/s12903-022-02468-9)
Supplement: Supplementary file 1 — Supplementary Tables [file 12903_2022_2468_MOESM1_ESM.docx]

Supplementary table 1. VAS pain score for patients in MHO group

| Patient No. | Gender | | Age | | Tooth | Doctor of tooth extraction | Dressing times | Pain score (VAS) | | | | | | |
| --- | --- | --- | --- | --- | --- | --- | --- | --- | --- | --- | --- | --- | --- | --- |
|  |  |  |  |  |  |  |  | 0h | 8h | 24h | day3 | day5 | day7 | day14 |
| 1 | | Male | | 27 | 48 | B | 1 | 7 | 6 | 2 | 0 | 0 | 0 | 0 |
| 2 | | Female | | 70 | 36 | C | 2 | 8 | 5 | 5 | 1 | 0 | 0 | 0 |
| 3 | | Female | | 61 | 23 | A | 1 | 9 | 5 | 4 | 1 | 0 | 0 | 0 |
| 4 | | Male | | 69 | 37 | A | 1 | 7 | 5 | 4 | 2 | 0 | 0 | 0 |
| 5 | | Male | | 28 | 38 | C | 1 | 7 | 3 | 5 | 0 | 0 | 0 | 0 |
| 6 | | Female | | 29 | 15 | C | 2 | 8 | 6 | 5 | 4 | 1 | 2 | 0 |
| 7 | | Female | | 27 | 48 | A | 1 | 8 | 3 | 2 | 1 | 0 | 0 | 0 |
| 8 | | Female | | 23 | 36 | C | 1 | 7 | 4 | 4 | 1 | 0 | 0 | 0 |
| 9 | | Female | | 29 | 36 | A | 1 | 9 | 5 | 3 | 1 | 0 | 0 | 0 |
| 10 | | Female | | 27 | 48 | A | 1 | 8 | 4 | 0 | 0 | 0 | 0 | 0 |
| 11 | | Male | | 67 | 46 | C | 1 | 8 | 3 | 1 | 0 | 0 | 0 | 0 |
| 12 | | Female | | 25 | 12 | A | 1 | 8 | 4 | 0 | 0 | 0 | 0 | 0 |
| 13 | | Female | | 31 | 48 | C | 1 | 10 | 7 | 4 | 1 | 0 | 0 | 0 |
| 14 | | Female | | 49 | 38 | C | 2 | 10 | 5 | 5 | 3 | 0 | 0 | 0 |
| 15 | | Female | | 31 | 38 | A | 1 | 8 | 6 | 4 | 0 | 0 | 0 | 0 |
| 16 | | Female | | 36 | 48 | C | 1 | 6 | 3 | 3 | 0 | 0 | 0 | 0 |
| 17 | | Male | | 29 | 48 | A | 1 | 7 | 4 | 0 | 0 | 0 | 0 | 0 |
| 18 | | Female | | 38 | 48 | A | 1 | 8 | 4 | 0 | 0 | 0 | 0 | 0 |
| 19 | | Female | | 67 | 48 | B | 1 | 7 | 4 | 4 | 2 | 0 | 0 | 0 |
| 20 | | Male | | 37 | 48 | C | 1 | 8 | 4 | 4 | 0 | 0 | 0 | 0 |
| 21 | | Male | | 33 | 38 | C | 2 | 7 | 5 | 5 | 4 | 0 | 0 | 0 |
| 22 | | Female | | 30 | 38 | C | 3 | 8 | 5 | 5 | 4 | 4 | 0 | 0 |
| 23 | | Male | | 26 | 38 | B | 1 | 10 | 5 | 5 | 0 | 0 | 0 | 0 |
| 24 | | Male | | 41 | 18 | C | 1 | 8 | 3 | 0 | 0 | 0 | 0 | 0 |
| 25 | | Male | | 22 | 38 | C | 2 | 9 | 5 | 5 | 4 | 2 | 0 | 0 |
| 26 | | Female | | 56 | 47 | B | 2 | 8 | 6 | 5 | 3 | 0 | 0 | 0 |
| 27 | | Male | | 31 | 48 | C | 1 | 7 | 4 | 2 | 1 | 0 | 0 | 0 |
| 28 | | Female | | 64 | 37 | A | 1 | 8 | 4 | 2 | 2 | 0 | 0 | 0 |

A. junior doctor in our department; B. senior doctor in our department; C. doctor in other places

Supplementary table 2. VAS pain score for patients in IG group

| Patient No. | Gender | | Age | | Tooth | Doctor of tooth extraction | Dressing times | Pain score (VAS) | | | | | | |
| --- | --- | --- | --- | --- | --- | --- | --- | --- | --- | --- | --- | --- | --- | --- |
|  |  |  |  |  |  |  |  | 0h | 8h | 24h | day3 | day5 | day7 | day14 |
| 1 | | Male | | 45 | 38 | C | 1 | 6 | 5 | 4 | 3 | 0 | 0 | 0 |
| 2 | | Female | | 41 | 36 | A | 1 | 7 | 5 | 5 | 3 | 0 | 0 | 0 |
| 3 | | Male | | 26 | 48 | C | 1 | 8 | 7 | 7 | 3 | 2 | 1 | 0 |
| 4 | | Male | | 31 | 38 | B | 1 | 8 | 7 | 6 | 2 | 1 | 0 | 0 |
| 5 | | Female | | 63 | 47 | C | 1 | 8 | 5 | 5 | 1 | 0 | 0 | 0 |
| 6 | | Female | | 28 | 48 | C | 2 | 10 | 8 | 7 | 5 | 1 | 0 | 0 |
| 7 | | Male | | 32 | 38 | C | 1 | 9 | 7 | 5 | 3 | 3 | 1 | 0 |
| 8 | | Female | | 20 | 38 | C | 2 | 8 | 7 | 7 | 5 | 4 | 2 | 0 |
| 9 | | Female | | 39 | 38 | C | 1 | 8 | 7 | 5 | 2 | 2 | 0 | 0 |
| 10 | | Female | | 21 | 38 | C | 2 | 9 | 6 | 6 | 4 | 3 | 2 | 0 |
| 11 | | Female | | 30 | 48 | C | 3 | 10 | 8 | 8 | 6 | 5 | 3 | 0 |
| 12 | | Female | | 49 | 48 | C | 2 | 9 | 8 | 6 | 5 | 3 | 2 | 0 |
| 13 | | Male | | 24 | 48 | C | 2 | 8 | 8 | 6 | 5 | 3 | 2 | 0 |
| 14 | | Female | | 31 | 47 | C | 1 | 8 | 6 | 5 | 3 | 3 | 0 | 0 |
| 15 | | Male | | 29 | 38 | B | 1 | 7 | 7 | 5 | 3 | 3 | 1 | 0 |
| 16 | | Female | | 27 | 38 | A | 2 | 7 | 7 | 6 | 4 | 2 | 1 | 0 |
| 17 | | Female | | 37 | 38 | C | 1 | 8 | 7 | 5 | 2 | 0 | 0 | 0 |
| 18 | | Female | | 25 | 38 | C | 1 | 8 | 6 | 5 | 3 | 1 | 0 | 0 |
| 19 | | Male | | 19 | 48 | C | 3 | 9 | 8 | 7 | 6 | 4 | 3 | 1 |
| 20 | | Male | | 34 | 34 | C | 2 | 8 | 6 | 5 | 4 | 3 | 1 | 0 |
| 21 | | Female | | 21 | 48 | C | 2 | 7 | 6 | 6 | 4 | 2 | 0 | 0 |
| 22 | | Male | | 39 | 48 | A | 1 | 7 | 5 | 3 | 0 | 0 | 0 | 0 |
| 23 | | Male | | 38 | 38 | B | 1 | 8 | 6 | 4 | 1 | 1 | 0 | 0 |

A. junior doctor in our department; B. senior doctor in our department; C. doctor in other places

Supplementary table 3. Adverse reactions of the treatment for AO

|  | Total  (N=51) |  | MHO Group  (N=28) |  | IG Group  (N=23) |
| --- | --- | --- | --- | --- | --- |
|  | Case number |  | Case number |  | Case number |
| Aggravated pain | 4 |  | 4 |  | 0 |
| Allergic reaction | 0 |  | 0 |  | 0 |
| Bleeding | 0 |  | 0 |  | 0 |
| Aggravated infection | 0 |  | 0 |  | 0 |
| Fever | 0 |  | 0 |  | 0 |
